# Understanding Maternal Role in Caring for Children with Severe Cognitive Impairment in Paediatric Palliative Care: A Qualitative Pilot Study

**DOI:** 10.3390/children13010119

**Published:** 2026-01-13

**Authors:** Anna Santini, Anna Marinetto, Danai Papadatou, Franca Benini

**Affiliations:** 1 Pediatric Pain and Palliative Care Service, Department of Women’s and Children’s Health, University Hospital, 35128 Padua, Italy; anna.santini@aopd.veneto.it; 2 Healthcare Profession Department, Padua University Hospital, 35128 Padua, Italy; anna.marinetto@aopd.veneto.it; 3 Department of Medicine, University of Padua, 35128 Padua, Italy; 4 Department of Mental Health and Behavioural Studies, Faculty of Nursing, National and Kapodistrian University of Athens, 10679 Athens, Greece; dpap@nurs.uoa.gr; 5 Department of Women’s and Children’s Health, University of Padua, 35128 Padua, Italy

**Keywords:** paediatric palliative care, maternal identity, severe cognitive impairment, mother–child bond, qualitative research, meaning making, disability caregiving, role construction

## Abstract

**Highlights:**

**What are the main findings?**

- The mother–child relationship in PPC is characterised by embodied attunement, fusion-like relational dynamics, and reliance on minimal non-verbal reciprocity.
- Mothers redefine their role through emotional labour, specialised interpretive caregiving, and meaning-making processes that support adaptation in the context of severe cognitive impairment.

**What are the implications of the main findings?**

- Recognising these relational and identity-related dynamics can help clinicians interpret non-verbal cues more accurately and engage mothers in more attuned, supportive communication.
- The findings highlight the need for personalised, meaning-oriented interventions and offer guidance for interdisciplinary teams working with families in PPC settings.

**Abstract:**

**Background/Objectives**: Within Paediatric Palliative Care (PPC), motherhood in the context of severe cognitive impairment is shaped by unique emotional, relational, and identity-related challenges. Traditional understandings of maternal identity are strained when verbal communication and typical developmental milestones are absent. Although caregiving in PPC has been widely studied, the subjective and symbolic dimensions of motherhood in this setting have received far less attention. This study sought to explore how mothers construct, interpret, and make sense of their maternal identity while caring for a child with severe cognitive impairment in a PPC context, and to underscore the clinical relevance of these identity-related processes. **Methods**: A qualitative study was conducted involving nine mothers of children receiving paediatric palliative care services at a regional centre in Italy. Participants engaged in three online focus groups, totalling 270 min. Reflexive thematic analysis was employed to interpret the transcribed data, using ATLAS.ti software, version 25.0.1 ATLAS.ti Scientific Software Development GmbH, Berlin, Germany, for support. Member reflections were incorporated to validate the findings. **Results**: Three interconnected themes emerged from the reflexive thematic analysis. First, mothers described the development of a fusion-like, enmeshed mother–child relationship, characterised by embodied attunement, specialised interpretive expertise, and lifelong care dependency. Second, mothers detailed the construction of their maternal role, shaped by emotional labour, identity negotiation, sacrifice, loneliness, and peer support, alongside the construction of the child’s role, in which children were perceived as unique, symbolically meaningful beings whose social presence and limited reciprocity shaped maternal identity. Third, mothers articulated a search for meaning that sustained them throughout the caregiving journey, reframing their experience within a broader existential and relational perspective. **Conclusions**: Maternal caregiving in PPC encompasses distinct emotional, relational, and symbolic dimensions that extend beyond conventional understandings of motherhood. Grasping these identity-related dynamics has direct clinical relevance: it enables more attuned communication, strengthens the therapeutic alliance, and supports personalised, meaning-oriented care. These insights highlight the need for tailored interventions and further qualitative research to inform health care professionals and interdisciplinary practice.

## 1. Introduction

Motherhood is not merely a biological event but a complex, multidimensional process shaped by relational, social, and cultural factors. The relationship with one’s child plays a central role in this journey, fostering a reciprocal emotional exchange that contributes to the construction of maternal identity. When a child presents with severe cognitive impairment, their ability to communicate and engage with the external world is significantly limited, thereby challenging the normative frameworks upon which maternal selfhood is typically constructed.

The maternal role, as conceptualised through Berger and Luckmann’s social construction theory of reality [1] and Goffman’s dramaturgical perspective [2], emerges as a dynamic performance enacted in everyday life. Mothers of children with profound cognitive disabilities must navigate societal expectations of idealised motherhood while responding to the concrete demands of their caregiving context. This performance involves symbolic tools—such as language, gestures, and appearance—that help sustain a coherent maternal self-image.

The social construction of motherhood comprises three interrelated processes: typification, institutionalisation, and internalisation. Typification refers to the shared expectations and behaviours that define the notion of a “good mother”, which becomes particularly complex in the context of disability caregiving. Institutionalisation reflects the influence of legal, educational, and cultural norms on maternal identity, while internalisation involves the integration of these societal expectations into the mother’s self-concept [3].

Psychoanalytic theories have historically illuminated the mother–child relationship. Winnicott introduced the concept of the “sufficiently good mother” [4]; Bowlby and Ainsworth developed attachment theory [5,6]; and Stern highlighted affective syntonisation [7]. However, these frameworks are difficult to apply when developmental milestones are unpredictable and emotional attunement is disrupted, as often occurs in cases of severe cognitive impairment.

Mothers of children with profound disabilities frequently experience grief for the imagined child, social isolation, and chronic caregiving strain. Despite these challenges, many develop resilience through emotion-focused and problem-solving coping strategies. Psychological support, peer networks, and ritualised practices can facilitate adaptation and well-being [8,9,10]. In paediatric palliative care (PPC) settings, the processes through which mothers construct and sustain their maternal identity while caring for a child with severe cognitive impairment remain largely unexplored. This gap persists because most studies prioritise clinical or practical aspects of caregiving over the symbolic and relational dimensions of motherhood that emerge under conditions of minimal reciprocity [11,12,13,14].

Given these conceptual, relational, and symbolic complexities, it is essential to enhance our understanding of how mothers shape and manage their maternal identity while caring for a child with severe cognitive impairment in a PPC setting. This research uniquely contributes to the field by investigating how mothers define their roles in fusion-like or enmeshed mother–child relationships in PPC, a topic that has not been previously studied.

The aim of this study is to explore the subjective meanings, identity-related processes, and relational dynamics through which mothers interpret and perform their maternal role under these conditions. By examining how maternal identity is reconstructed in the absence of verbal reciprocity and predictable developmental trajectories, this study seeks to illuminate caregiving dimensions largely overlooked in current PPC literature and to generate insights directly relevant to clinical practice. The scarcity of empirical research specifically addressing maternal identity in the context of severe cognitive impairment—supported only indirectly by broader reviews such as Pelentsov et al. [11], which further justifies the exploratory, pilot design of this study. This approach allows us to generate preliminary conceptual insights that can guide future, more extensive PPC research.

## 2. Materials and Methods

### 2.1. Study Design

This qualitative pilot study employed focus groups to explore maternal role construction among mothers of children with severe cognitive impairment receiving paediatric palliative care. The focus-group format was especially effective, as group interaction encouraged shared reflection, resonance, and the co-creation of meaning. This enabled mothers to build on one another’s stories and produce rich, multilayered data.

The manuscript conforms to COREQ criteria and includes explicit mapping of methodological rigour, reflexivity, and participant engagement (see Supplementary File S1 for the COREQ checklist) [15].

This study was informed by a constructivist–interactionist epistemology, which conceptualises maternal identity as a dynamic and relational construct shaped through situated meaning-making practices. Within this perspective, caregiving is not merely a behavioural response but a narrative process through which mothers interpret, negotiate, and embody their role in the context of profound cognitive impairment. Drawing on Bruner’s theory of narrative identity, maternal selfhood is understood as a culturally mediated story that organises experience and confers coherence to relational roles [16]. Gergen’s relational constructionism further supports this view by framing identity as dialogically sustained and embedded within social interaction [17]. These frameworks position mothers as active agents in the co-construction of meaning, rather than passive recipients of normative expectations.

To ensure epistemological congruence, reflexive thematic analysis was employed following Braun and Clarke’s approach [18,19]. This method acknowledges the researcher’s interpretative role and supports an iterative, theory-sensitive engagement with participant narratives. Themes were not treated as latent structures within the data but as constructed patterns shaped through reflexive dialogue and contextual understanding.

This framework enabled a nuanced interpretation of maternal role development, highlighting how identity is continuously redefined through emotional labour, symbolic negotiation, and relational positioning within paediatric palliative care.

### 2.2. Study Setting and Recruitment

The research was conducted at the Regional Centre for Paediatric Palliative Care and Pain Therapy, within the Department of Women’s and Children’s Health at the University Hospital of Padua, Italy. Participants were recruited from the paediatric hospice affiliated with the centre. Recruitment was conducted via telephone, followed by written information outlining the study’s aims, procedures, and ethical safeguards.

### 2.3. Participants’ Recruitment and Sampling

The study involved nine mothers of children who experience severe cognitive impairment and are receiving care at the paediatric hospice. The nine parents who met the eligibility criteria accepted to participate in the study by giving their consent.

No participants refused to take part or dropped out of the study. All individuals who were invited agreed to participate and completed the interview.

The eligibility and inclusion criteria were: (a) biological and legal mothers who possess intact parental responsibility; (b) mothers with a proficient understanding of both spoken and written Italian; and (c) mothers or patients with severely impaired intellectual and psycho-social functioning, according to items 177 and 122 of the International Classification of Functioning, Disability, and Health for Children and Youth. This classification is a conceptual model that describes health, functionality, and disability, providing guidelines for assessing children’s capacity to perform activities drawn from a predetermined list. It represents a standardised tool for evaluating the child’s overall functioning on a 5-point scale, where 0 signifies no impairment, and 4 indicates total disability [20]. Item b-117 assesses the capacity to constructively understand and integrate various mental functions, including all cognitive functions and their development over a lifetime. In contrast, Item b-122 assesses the mental functions that are required to understand and constructively integrate mental tasks that form the interpersonal skills necessary to establish reciprocal social interactions, meaning, and purpose.

The ICF-CY classification was developed by a team of paediatric palliative care specialists who were intimately familiar with the patient population. Initially, two groups, each comprising two authors, conducted an assessment, which was subsequently shared in a plenary session to ensure a clear and concise evaluation.

### 2.4. Data Collection

Participants were selected through purposive sampling, based on predefined clinical and linguistic inclusion criteria, to ensure the relevance of their experiences to the study’s aims and to capture rich, context-specific insights. Recruitment was conducted via telephone, followed by written information outlining the study’s objectives, procedures, and ethical safeguards. Informed consent was obtained prior to participation, and confidentiality and voluntary involvement were ensured.

There were no pre-existing relationships between researchers and participants. Before each session, the researchers—two female healthcare professionals with expertise in paediatric palliative care and qualitative methods—clarified their roles and the study’s purpose to minimise relational bias. Both researchers were employed in public healthcare settings and had several years of clinical and research experience. One was a nurse who had completed training courses in qualitative research methods, while the other was a psychologist with specialised training in qualitative research software. Participants were aware of the researchers’ professional roles and institutional affiliations, but no personal information—such as individual goals or reasons for conducting the study—was disclosed to reduce potential bias. The facilitators of the interviews were two of the authors, both serving as researchers.

All interviews were conducted via secure video conferencing platforms, with participants connecting from private settings in their homes. No third parties were present during the sessions. Each of the nine mothers participated in all three online focus group sessions (90 min each; totalling 270 min), which fostered rapport and allowed for thematic saturation. Data collection was discontinued when no new conceptual elements emerged.

The discussions were facilitated by the two researchers, whose role was to guide the conversation and provide space for participants to share their experiences. The interview approach was shaped by the researchers’ clinical experience and preceded by a collaborative brainstorming session among the authors. This preparatory phase helped refine the interview prompts and ensure alignment with the study’s theoretical framework, which was grounded in narrative and constructivist paradigms. Core guiding questions included:

- “What aspects of being a mother move you emotionally?”
- “What is sacred to you in your relationship with your child?”
- “What gives meaning to your relationship with your child?”

All sessions were audio-recorded with consent and transcribed verbatim. During and immediately after each session, the researchers took field notes to document contextual observations, non-verbal cues, and initial impressions. No repeat interviews were conducted.

### 2.5. Data Analysis

The qualitative data were analysed using reflexive thematic analysis as outlined by Braun and Clarke [18]. This method allows for the identification and interpretation of meaningful patterns while emphasising the researcher’s subjectivity and reflexivity. The process followed six iterative phases: familiarisation with the data, initial coding, theme development, theme review, theme definition and naming, and the production of the final report.

Two researchers attended the focus group sessions and were subsequently involved in the analysis. One researcher (AS), with a background in clinical psychology, transcribed the data verbatim, while the second (AM), trained in nursing, revised the transcript and contributed to theme coding. Both researchers have over six years of professional experience in paediatric palliative care. The analysis was conducted reflexively, with regular peer discussions to support interpretative depth and analytical coherence. Coding was performed collaboratively, allowing for triangulation and enhancing the reliability of the thematic structure. To enhance analytic rigour, we employed an iterative coding process, maintained a reflexive stance throughout the analysis, and ensured transparent theme development in line with established criteria for trustworthiness in qualitative research.

The coding process was inductive and supported by the qualitative analysis software ATLAS.ti. A preliminary thematic framework was presented to participants for member reflection, who confirmed that the findings resonated with their experiences and did not suggest substantial changes. This step fostered transparency and reflexive engagement, in line with the study’s constructivist epistemology.

Additionally, sociodemographic information of participants and patients was collected and analysed descriptively, including means, medians, and standard deviations.

Transcripts were returned to participants to interpret findings, review thematic domains, and validate implications for practice, ensuring the research addressed caregiver-relevant priorities.

## 3. Results

In this section, we present the findings of the socio-demographic and qualitative analysis. The data collected offer a comprehensive insight into the characteristics of mothers and their children with severe cognitive impairments, as well as the mothers’ experiences and perceptions of their maternal role.

### 3.1. Sociodemographic Findings

The sociodemographic analysis provides a comprehensive description of the study sample, including mothers’ age, marital status, educational level, and occupation, as well as the clinical characteristics of their children. This information aids in understanding the socio-economic and familial context within which mothers reside and provide care to their children.

#### 3.1.1. Characteristics of Mothers

The mothers participating in the study were on average 46.3 years old (SD 5.0). Most were married, and one was widowed. Educational levels ranged from high school diplomas to university degrees, and employment status varied from full-time to unemployed. Two mothers reported having no family or social support. A detailed overview is provided in Table 1.

#### 3.1.2. Characteristics of Patients

The mean age of the children was 13.8 years, with a standard deviation of 5.8 years. This diverse cohort also comprised patients exceeding the age of 18, as paediatric palliative care may be extended until the age of 23. Most had non-oncological conditions and presented with severe to complete impairments in intellectual and psychosocial functioning. Visual and auditory impairments varied across the sample, and six children had siblings. Full clinical characteristics are summarised in Table 2. The children’s clinical profiles reflected the high complexity typically encountered in PPC settings.

### 3.2. Qualitative Findings

The qualitative analysis of the focus group discussions offers an in-depth exploration of how mothers experience and make sense of their relationship with a child affected by severe cognitive and physical disabilities. This approach enabled the identification of subtle emotional, relational, and symbolic processes that shape maternal identity in the context of paediatric palliative care. Through reflexive thematic analysis, three overarching themes were developed:

- the mother–child fusion-like and enmeshed relationship and its evolution,
- the construction of the maternal role, and,
- the search for meaning that sustains mothers throughout their caregiving journey.

These themes were grounded in the participants’ narratives and are illustrated through selected verbatim quotations. Quotations are reported in single quotation marks and followed by an anonymised reference indicating the speaker and the focus group (e.g., Mother 3, FG1).

#### 3.2.1. Theme 1—The Mother–Child Fusional Bond and Its Evolution

**Figure 1:**
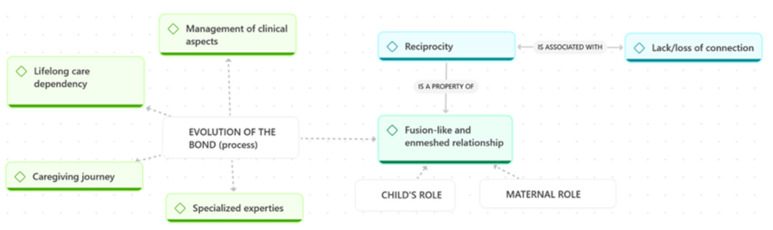
Theme 1: Forms and Transformations of the Mother–Child Relationship.

Theme 1 explores how mothers described the emotional and relational dynamics that shape their bond with their child. Drawing on the narratives of all nine participants, this theme captures both the evolving nature of the relationship and the deeply embodied, fusion-like connection that many mothers reported. Figure 1 provides a visual diagram of the thematic structure, illustrating the relationships between the codes that compose this theme.

##### Evolution of the Bond

Across the focus groups, mothers described the relationship with their child as a dynamic, continuously evolving process shaped by clinical fragility, emotional attunement, and the demands of long-term caregiving. As illustrated in Figure 1, this evolution of the bond unfolded through four interconnected dimensions: specialised expertise, management of clinical aspects, lifelong care dependency, and the broader caregiving journey that mothers experienced alongside their child.

Many participants emphasised the development of specialised expertise, referring to a refined ability to interpret subtle physiological cues, behavioural changes, and sensory signals. This interpretive competence became essential in contexts where verbal communication was limited or absent:

*‘Even though he doesn’t speak, we can still perceive his different types of breathing.’*

(Mother 4, FG2)

Closely linked to this expertise was the management of clinical aspects, which required mothers to integrate medical knowledge into everyday caregiving. Several participants described becoming highly skilled in monitoring symptoms, adjusting routines, and responding to clinical needs with precision and confidence, often performing tasks typically associated with trained professionals.

The development of the bond was also shaped by lifelong care dependency. Despite their child’s chronological age, mothers noted that daily care needs often resembled those of a very young infant, requiring constant presence, night-time monitoring, and sustained physical and emotional availability:

*‘I still wake up at night if she wakes up, like when there is a newborn baby.’*

(Mother 2, FG1)

Finally, many mothers framed their experience as an ongoing caregiving journey—a long-term, unfolding path marked by discovery, transformation, and shared meaning. This metaphor captured both the challenges and the profound sense of purpose that emerged over time:

*‘The journey with our children is really a journey, it is a continuous discovery.’*

(Mother 1, FG1)

Together, these four dimensions illustrate how the bond evolved not as a linear developmental trajectory, but as a continuous, adaptive interplay between clinical competence, emotional attunement, and enduring caregiving responsibilities.

##### Fusion-Like and Enmeshed Relationship

Alongside this evolving process, mothers described a fusion-like and enmeshed relationship characterised by profound emotional, physical, and intuitive closeness. Many participants portrayed the bond as deeply embodied, often blurring the boundaries between self and child. This visceral connection shaped their daily caregiving and emotional world:

*‘I feel and perceive every feeling she has… I still wake up at night if she wakes up.’*

(Mother 2, FG1)

Some mothers described this closeness in even more intimate terms, emphasising a sense of shared identity:

*‘I mean a visceral relationship… with her it is as if I live in her.’*

(Mother 2, FG1)

*‘We are one, he is a part of me.’*

(Mother 5, FG2)

While this closeness was often experienced as meaningful and protective, it could also become overwhelming. One mother reflected on the emotional cost of remaining constantly attuned:

*‘It becomes survival… I was too much with her, it didn’t make me live a bit.’*

(Mother 3, FG1)

Within this relational configuration, reciprocity emerged as a key property of the fusion-like bond. Even minimal or non-verbal cues—such as a smile, a change in breathing, or a subtle bodily signal—were experienced as meaningful forms of mutual recognition:

*‘Our children’s smiles repay us for all this.’*

(Mother 3, FG1)

However, mothers also described moments of lack or loss of connection, particularly when sensory impairments limited the child’s ability to respond. These episodes intensified emotional labour and highlighted the fragility of non-verbal reciprocity:

*‘She smiles, but who knows what goes through her mind?… we don’t really get anything back.’*

(Mother 4, FG1)

Together, reciprocity and lack/loss of connection illuminate the delicate balance that characterises fusion-like and enmeshed relationships. While embodied closeness can foster deep attunement, the absence of reciprocal cues can heighten uncertainty and emotional strain, underscoring the complexity of caregiving amid severe cognitive and sensory impairments.

#### 3.2.2. Theme 2—Role Construction

**Figure 2:**
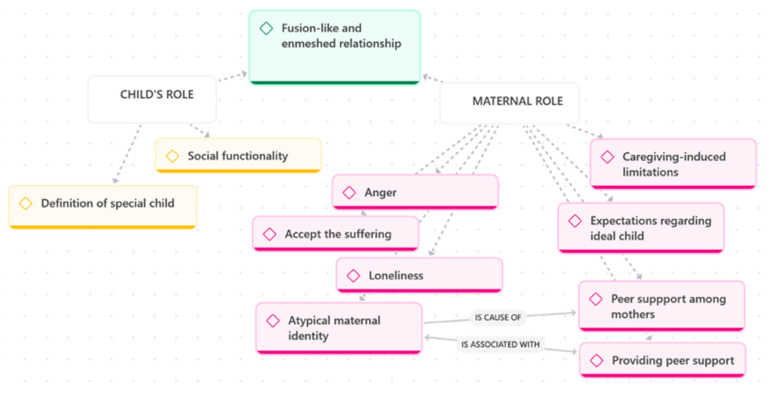
Maternal and Child Roles.

Theme 2 explores how mothers construct and negotiate the roles that define their relationship with their child within fusion-like and enmeshed relational dynamics. As illustrated in Figure 2, role construction unfolds along two interconnected dimensions: the maternal role, shaped by emotional labour, identity negotiation, and peer support; and the child’s role, shaped by how mothers define their child’s uniqueness and interpret their social presence and functionality. Together, these dimensions reveal how mothers make sense of their caregiving experience and the relational positions they inhabit.

##### Maternal Role

The construction of the maternal role is intrinsically connected to the experience of motherhood when caring for a child with significant cognitive and physical disabilities. Mothers described enduring numerous sacrifices, facing deprivation, and experiencing profound loneliness. A strong perception of being distinct from other mothers emerged, often leading to a sense of isolation:

*‘Before, maybe I even had some anger in it, the fact of why did this happen to me.’*

(Mother 3, FG1)

*‘It’s constant sacrifice, it’s a capital M getting bigger and bigger to try to make them feel good.’*

(Mother 3, FG1)

Anger and loneliness were persistent emotions, coupled with the necessity to acknowledge suffering as a fundamental aspect of their child’s life:

*‘Because at a certain point, it was no longer controllable, and the difficult thing is not to accept the disability, given that we were used to it, but to accept the suffering.’*

(Mother 4, FG2)

A central component of the maternal role was the emergence of an atypical maternal identity, a sense of being a mother who is fundamentally different from others due to the intensity and complexity of caregiving. This identity was often described as possessing an “extra edge”:

*‘Oh God, I don’t want to be considered Wonder Woman, but having a son who is well is easy.’*

(Mother 3, FG1)

The maternal role also involved confronting expectations regarding the ideal child, a process that required mothers to let go of imagined futures and reorient their identity around their child’s actual needs and capacities:

*‘You idealise it when you have it in your belly and hope it will be OK… the important thing is that it’s fine.’*

(Mother 3, FG1)

Experiences of stigma and misunderstanding further shaped this atypical identity:

*‘However, with the world of the “normal” people, there is really sometimes a wall and not even a will to dialogue, to understand who is on the other side, what they are feeling.’*

(Mother 3, FG1)

As shown in Figure 2, atypical maternal identity is a cause of the need for peer support among mothers. Connecting with other mothers in similar circumstances was described as essential for alleviating feelings of isolation and fostering mutual understanding:

*‘And sometimes you kind of find yourself… sharing things with the other moms—realising that there are so many people going through the same things, and you connect even without saying much… sometimes just a look is enough to understand each other.’*

(Mother 1, FG1)

This identity was also associated with a strong desire to provide peer support to others, expressed as a mission to help mothers who were earlier in the journey:

*‘Sharing and knowing that there are other people who’ve felt what you’ve felt—and being understood—really helps… maybe we can also give something back in return.’*

(Mother 2, FG1)

##### Child’s Role

In parallel with constructing their own maternal role, mothers articulated a distinct child’s role, shaped by how they defined their child’s uniqueness and interpreted their child’s social presence. As illustrated in Figure 2, this dimension comprises two key processes: defining the special child and the child’s social functioning.

##### Definition of the Special Child

Mothers frequently described their children as “special beings”, emphasising their uniqueness, depth, and symbolic significance. This definition transcended clinical labels and highlighted the child’s emotional and existential presence within the family:

*‘Challenging, special, unique in its style… really it always has a new chapter every day; you never know what you will find, what awaits you.’*

(Mother 1, FG1)

Some mothers articulated this specialness in explicitly spiritual or philosophical terms, describing their children as possessing an inner life that exceeded their physical limitations:

*‘To me, they’re special beings… souls trapped in a body. And a soul is made of memory, intellect, and will… even if it might look like they don’t perceive or understand… we know it’s not like that, because we live it every day. I live my daughter’s disability as a gift.’*

(Mother 1, FG1)

##### Social Functionality

Mothers also reflected on their child’s social functioning, describing how their presence could transform others’ perceptions and attitudes. Many saw their children as catalysts for positive change, capable of eliciting empathy, reflection, and personal growth in those who encountered them:

*‘I am convinced that these boys and girls, children, have come to bring out the best in each of us.’*

(Mother 3, FG1)

For some mothers, the child’s social role extended beyond immediate interactions and became a form of legacy, carried forward through storytelling and public presence:

*‘For me, something that can truly be sacred is the act of telling S.’s story—of making her known… bringing her life into the world through storytelling. For me, her presence is something sacred because I can still honour her presence.’*

(Mother 3, FG3)

Through these narratives, the child’s role emerges as both symbolic and relational: children are positioned as unique individuals whose presence shapes family identity, while also holding a transformative social value that reorients perceptions and reshapes relational dynamics.

#### 3.2.3. Theme 3—Search for Meaning

**Figure 3:**
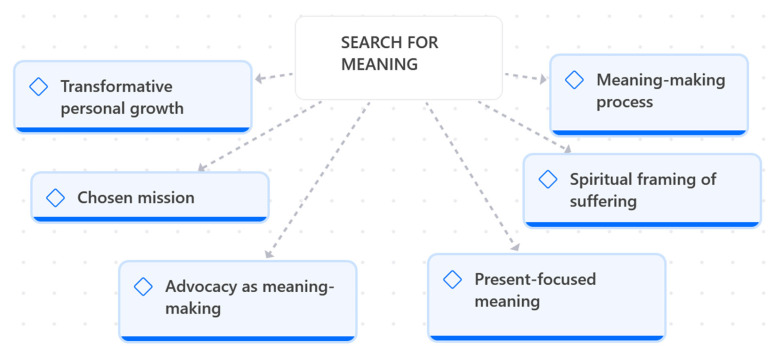
Search for meaning.

Theme 3 explores how mothers engage in a multifaceted meaning-making process to interpret and sustain their caregiving experience. As illustrated in Figure 3, this process unfolds through intertwined dimensions of transformative personal growth, advocacy, and meaning-making, and the emergence of a chosen mission.

Many mothers described their journey as one of profound inner evolution, marked by emotional maturation, resilience, and a reorientation of personal values. Caring for a child with severe disabilities was portrayed not only as a challenge but as a catalyst for becoming a different version of themselves:

*‘I have grown so much… I am not the person I was before; she has changed me in ways I never imagined.’*

(Mother 2, FG1)

This transformation often translated into a strong commitment to advocacy, through which mothers fought for recognition, rights, and dignity for their children. Advocacy became a way to transform frustration into purpose, reinforcing the sense that their actions carried meaning beyond daily caregiving:

*‘You have to fight every day… for services, for understanding, for respect. And in that fight, you find meaning—you feel you’re doing something important.’*

(Mother 4, FG2)

Over time, many mothers reframed their role as a chosen mission, a deliberate and meaningful commitment that grounded their identity and sustained them through uncertainty:

*‘At a certain point, you choose it… you choose to be there, to do it, to carry it forward. It becomes your mission.’*

(Mother 1, FG1)

Alongside these processes, mothers cultivated a present-focused meaning, emphasising the importance of living day by day and finding value in small gestures and shared moments:

*‘We live day by day… we don’t think too far ahead. What matters is today, what she gives us today.’*

(Mother 5, FG2)

For some, the search for meaning also took on a spiritual framing of suffering, allowing them to attribute sacredness, depth, or existential value to their child’s condition and to their own emotional pain:

*‘Her suffering… I see it as something sacred, something that teaches us. It’s painful, yes, but it has a meaning.’*

(Mother 3, FG1)

Across the focus groups, mothers described an ongoing meaning-making process that helped them integrate emotions, reinterpret adversity, and construct a coherent narrative of their lives with their child:

*‘You look for meaning in everything… in what happens, in what you feel, in what they teach you. Otherwise, you wouldn’t make it.’*

(Mother 6, FG2)

Together, these dimensions reveal how meaning becomes a central psychological and existential resource, enabling mothers to navigate complexity, sustain hope, and anchor their caregiving experience within a broader sense of purpose.

## 4. Discussion

This qualitative study enriches the growing body of work on parenting in paediatric palliative care by illustrating how caring for a neurologically complex child reshapes the maternal role well beyond what the mainstream attachment and disability literature has described to date. Consistent with this, our cohort lived in “high-care” households coping with daily medical complexity. Unlike most paediatric palliative studies that focus on single-child families, over half of our mothers also parent typically developing siblings who themselves need to make sense of disability [21,22,23,24]. This confirms the “role-strain” pattern reported in previous work [8]. However, we extend these findings by showing that employment outside the home adds a layer of identity conflict for more than half of the participants, even when informal support networks are present. The persistence of self-reported isolation despite support echoes—and therefore strengthens—the recent findings of Brekke & Alecu [10], suggesting that loneliness is a cross-cultural constant rather than a context-specific artefact.

Our data corroborate Mahler’s classic concept of an early “fusion” phase [25], yet contradict her expectation of progressive separation–individuation in later childhood. Mahler conceptualised early fusion as a normative stage grounded in reciprocal cues such as gaze, vocalisation, and early exploratory behaviours. In our cohort, mothers indeed described a visceral, embodied closeness consistent with this early phase; however, the expected developmental shift towards differentiation does not occur because children lack the communicative and motor indicators that typically scaffold separation. Like Stern [7], we found that reciprocity can flourish without speech, but we add nuance by showing that sensory–motor impairment does not preclude such reciprocity; rather, mothers refine new channels (tactile micro-cues, bio-medical data, intuition). This suggests that reciprocity in profound disability is constructed through alternative, non-classical indicators, expanding Stern’s model of affective attunement. This extension of Stern’s thesis aligns with Ainsworth’s and Bowlby’s stress-buffering models [5,6], but challenges the assumption that attachment quality inevitably declines when verbal interaction is limited. Taken together, these findings call for theoretical models that account for non-normative developmental trajectories and embodied forms of communication.

Emotionally, mothers described experiences of anger, loneliness, and sustained sacrifice. These emotions were not merely indicators of burden; prior research shows that they often become part of the identity-shaping process in families of children with severe disabilities [26,27,28]. In the absence of conventional developmental trajectories and reciprocal cues, mothers were required to reinterpret their responsibilities and redefine what it means to be a mother to a child with profound disabilities, a process consistent with earlier work on maternal identity reconstruction [29]. Through this process, emotional strain became intertwined with identity formation, supporting the development of an atypical maternal identity that integrates vulnerability, commitment, and resilience. This identity work was closely connected to how mothers perceived their children. Rather than viewing them solely through the lens of impairment, mothers described their sons and daughters as “special beings,” whose presence held symbolic and relational significance. This reframing echoes philosophical and sociological accounts of the moral and transformative value of disability [30,31] and aligns with Robinson et al. [32], who argue that disabled children can become moral change agents. In our study, mothers described their children as catalysts for empathy, sensitivity, and advocacy, suggesting that the maternal role is sustained not only by caregiving demands but also by a sense of purpose grounded in the child’s perceived transformative value.

This meaning-making process—encompassing transformative personal growth, present-focused living, and spiritual framing of suffering—highlights how mothers construct coherence and purpose in the face of profound caregiving demands. Such processes resonate with Bruner’s narrative identity theory [16] and Gergen’s relational constructionism [17], and are consistent with broader existential perspectives that conceptualise meaning as a central resource in contexts of suffering [33]. The link between meaning and resilience observed in our cohort also aligns with findings from caregiving research, which show that benefit-finding and purpose attribution can buffer psychological distress [34]. Together, these insights underscore that maternal identity in paediatric palliative care is not merely a behavioural role but a symbolic, relational, and existential project.

### 4.1. Strengths and Limitations of the Work

The principal strength of this study is its capacity to illuminate the otherwise invisible micro-practices through which mothers of children with severe neurological impairment continuously decode clinical cues and convert them into tailored caregiving actions. By documenting nocturnal anxieties, improvised bedside routines, and sustained advocacy behaviours, the analysis foregrounds a reservoir of tacit clinical knowledge rarely captured in medical records yet central to the child’s comfort, dignity, and prognosis; few studies capture day-to-day embodied caregiving knowledge [8,9,10,32].

This study presents several limitations that may affect the transferability and generalisability of its findings. First, the sample was small (*n* = 9) and culturally homogeneous, consisting exclusively of Italian, Catholic, heterosexual mothers. This restricts the applicability of the results to more diverse populations, including fathers, non-parent caregivers, and families from different cultural or religious backgrounds. Second, data collection was conducted via online focus groups. While this format facilitated participation, it may have limited the depth of emotional expression and interpersonal connection. As Yin cautions, virtual settings can dampen rapport and reduce the richness of qualitative data, particularly when exploring sensitive or emotionally charged topics [35]. Third, the study relied on self-reported narratives, which are inherently subjective and may be influenced by social desirability or retrospective bias. Finally, the cross-sectional design captures maternal experiences at a single point in time, without accounting for potential changes in role perception or coping strategies that may occur across the child’s developmental trajectory.

### 4.2. Recommendations for Further Research

Future studies should replicate these findings across culturally diverse and structurally varied family systems. Inclusion of fathers, grandparents, and non-parent caregivers would enrich the understanding of caregiving dynamics. Longitudinal, in-person designs are recommended to explore how maternal role perception and advocacy trajectories evolve over time. Moreover, research should examine the timing and impact of interventions aimed at fostering relational boundaries and supporting the development of maternal identity.

### 4.3. Implications for Policy and Practice

The findings suggest that clinicians should reconsider the timing of psychosocial interventions, prioritising boundary-building support earlier in the caregiving journey. Training programmes for paediatric palliative care professionals should incorporate modules on maternal identity negotiation, emotional labour, and tacit caregiving knowledge. Policies should recognise mothers as clinical partners and moral agents, whose experiential expertise contributes meaningfully to care planning and decision-making.

By reframing adversity as a catalyst for spiritual growth and outward-facing activism, mothers in this study endorsed a sacred narrative arc that complements existing models of advocacy. This reframing has implications for how healthcare teams engage with families, suggesting the need for relationally attuned, meaning-sensitive approaches to care.

## 5. Conclusions

This pilot study demonstrates that mothers of children with severe cognitive disabilities in paediatric palliative care develop a fusion-like, boundaryless bond with their children, one that persists as the child grows and is shaped by both the child’s clinical fragility and the mother’s intimate, embodied understanding of it.

Future work will expand the sample to include culturally diverse mothers, fathers, and other caregivers, follow them longitudinally, and conduct face-to-face sessions to deepen relationships. Understanding how the maternal role evolves in paediatric palliative care is crucial for designing targeted support and for informing therapeutic decisions grounded in a richer, more reflective understanding of the mother–child relationship.

These insights also carry important clinical implications, highlighting the need for relationally attuned, meaning-sensitive approaches—integrated into routine PPC practice—that recognise mothers as active partners in care and support the ongoing construction of their maternal identity, thereby strengthening family-centred care, facilitating shared therapeutic decision-making, and helping clinicians understand the relational and emotional challenges that may shape a mother’s response to proposed interventions.

## Figures and Tables

**Table 1:** Profile of Participating Mothers.

Characteristics of the Mothers (*n* = 9)	
Age (years), mean +/− (SD) median	46.3 +/− 46 (5.0)
Marital status (*n*)	
Married	8
Widow	1
Educational background (*n*)	
High school	5
University degree	4
Professional occupation, number (*n*)	
Full-time	2
Part-time	3
Unemployed	4
Lack of family/social support (*n*)	2

**Table 2:** Overview of Patient Profiles.

Characteristic of Patients (*n* = 9)	
Age(years), mean +/− (SD) median	13.8 +/− 5.8 (16.0)
Diagnosis, number (*n*)	
oncological	0
non-oncological	8
undefined diagnosis	1
ICF-CY (*n*)	
Item b210: visual functioning impairment	
none	4
severe	0
complete	5
Item b230: auditory functioning impairment	
none	6
severe	2
complete	1
Item b177: intellectual status impairment	
severe	0
complete	9
Item b122: psycho-social impairment	
severe	5
complete	4
Presence of siblings (*n*)	6

## Data Availability

The datasets generated and analysed during the current study are not publicly available due to confidentiality agreements, but are available from the corresponding author on reasonable request.

## Supplementary Materials

The following supporting information can be downloaded at: https://www.mdpi.com/article/10.3390/children13010119/s1, The COREQ checklist is available as Supplementary Materials (File S1). Reference [15] are cited in supplementary materials

## Abbreviations

The following abbreviations are used in this manuscript:

PPC	Paediatric Palliative Care
